# Challenges in Treating Secondary Syphilis Osteitis in an Immunocompromised Patient with a Penicillin Allergy: Case Report and Review of the Literature

**DOI:** 10.1155/2016/4983504

**Published:** 2016-05-29

**Authors:** Robert Ali, Julio Perez-Downes, Firas Baidoun, Bashar Al Turk, Carmen Isache, Girish Mohan, Charles Perniciaro

**Affiliations:** ^1^Department of Internal Medicine, University of Florida-Jacksonville, 655 W 8th Street, Jacksonville, FL 32209, USA; ^2^Department of Pathology, University of Florida-Jacksonville, 655 W 8th Street, Jacksonville, FL 32209, USA

## Abstract

Syphilis is a sexually transmitted infection that remains fairly commonplace. The introduction of penicillin aided in curbing the incidence of disease; however, with the advent of the human immunodeficiency virus (HIV), syphilis is now on a resurgence with sometimes curious presentations. We present a case of a 36-year-old Caucasian gentleman with untreated HIV who complained of a skin eruption and joint pains for 6 weeks, prompting the diagnosis of secondary syphilis osteitis. Skin lesions were reminiscent of “malignant” syphilis. CD4 count was 57 cells/*μ*L. RPR was elevated with 1 : 64 titer and positive confirmatory TP-PA. Radiography of the limbs revealed polyostotic cortical irregularities corroborated on bone scintigraphy. The patient had an unknown penicillin allergy and was unwilling to conduct a trial of penicillin-based therapy. He was subsequently treated with doxycycline 100 mg twice daily for 6 weeks and commenced antiretroviral therapy, noting dramatic improvement in both the skin lesions and joint pains. Unfortunately, he defaulted on follow-up, precluding serial RPR and bone imaging. Penicillin allergies have proven to be quite a conundrum in such patients, without much recourse for alternative therapy. Doxycycline with/without azithromycin is other options worth considering.

## 1. Introduction

Syphilis is a sexually transmitted disease which is still prevalent in the world. It is caused by the spirochete* Treponema pallidum*, and it is transmitted mainly by contact with a syphilitic sore. These sores are most commonly present in the genitals and can be located both internally and externally [1]. Syphilis can be classified into several stages according to the clinical manifestations of the disease. These include early syphilis, which typically occurs within the first year of infection and can be divided into primary, secondary, and early latent syphilis, as well as latent syphilis, which is characterized as asymptomatic infection with a positive serology [2]. Other presentations of syphilis also include central nervous system involvement, causing neurosyphilis, as well as extensive vascular involvement with syphilitic aortitis [2].

Due to the advent of antibiotics and the increased awareness and health campaigns throughout the United States and the world, the incidence of syphilis has decreased significantly over the past decades [3]. In addition, better screening methods, early identification, and more access to healthcare have allowed early treatment, further reducing the clinical presentation, and diagnosis of the late manifestations of syphilis [3]. Despite such advancements, secondary syphilis is still prevalent in high risk populations, mainly individuals with the human immunodeficiency virus (HIV), people who engage in high risk sexual activity, and those who do not regularly use protection [3]. In this case, we present the uncommon finding of secondary syphilis in an immune-compromised individual, with diffuse bone involvement and concurrent allergies to penicillin, necessitating treatment with doxycycline.

## 2. Case

A 36-year-old Caucasian, homosexual male with known human immunodeficiency virus (HIV) infection for the past 15 years presented with a disseminated skin eruption over 6 weeks. The patient had been without antiretroviral medications for the past 8 years, defaulted on follow-up, and was uncertain of his last CD4 count or viral load.

**Figure 1:**
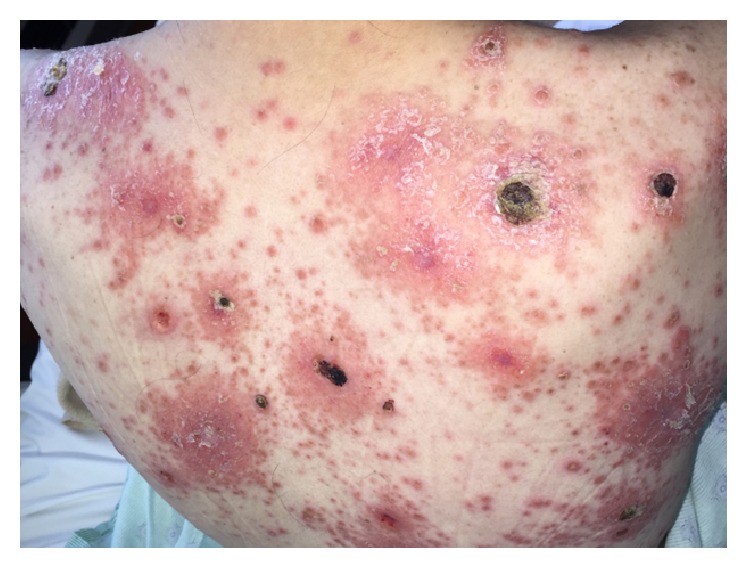
Photograph of the patient's back demonstrating an erythematous skin eruption with patches of desquamation, plaques, and papules.

The eruption was composed of annular, scalene papules and plaques, some of which were ulcerated (Figure 1). Lesions were distributed on the trunk, extremities, and face. A few lesions demonstrated a thick, ostraceous crust consistent with the skin eruption described in “malignant” syphilis [5].

Constitutional symptoms included night sweats, decreased appetite, and fatigue. The patient also complained of joint pains at both hands and feet. He denied recent travel, walks in the woods, and recent environmental changes. He could not recall any sick contacts.

Of note, he was allergic to penicillin. The allergy was ascribed since childhood and he could not recall the specific reaction but did emphasize that he was strongly advised against taking any penicillin-containing antibiotics. He denied recent sexual interaction but stated that he contracted HIV via anal intercourse.

The patient looked unwell on general examination. He had pink, but dry mucous membranes and was mildly tachycardic with otherwise unremarkable vital signs. White, adherent plaques were noted in the mouth. The diffuse skin eruption was noted as above. Joint swelling and tenderness were also noted at the wrist and ankles. Cardiovascular, respiratory, abdominal, and neurological examinations were noncontributory.

Initial hematologic workup was significant for mild pancytopenia, leukocyte count 3.4 × 10^3^/mm^3^, hemoglobin 10.2 g/dL, and platelet count 123 × 10^3^/mm^3^. The CD4 count was 57 cells/*μ*L and HIV RNA was 365,000 copies/mL. Antinuclear antibody and rheumatoid factor assays were both negative.

Evaluation for additional sexually transmitted infections revealed a negative acute hepatitis panel but a positive rapid plasma reagin (RPR) with a titer of 1 : 64. Confirmatory testing with* Treponema pallidum* antibody (TP-PA) testing was positive. A call to the department of health confirmed no previously reported syphilis infection. Subsequent lumbar puncture was not consistent with infection, specifically a negative VDRL. Blood cultures and wound swabs were negative, and the sedimentation rate was 132 mm/hr.

Punch biopsies were performed on the skin lesions at the left forearm and the left thigh. The histopathologic features were those of psoriasiform lichenoid dermatitis, typical for secondary syphilis. Plasma cells were not readily identified in the infiltrate. Because of the concern for syphilis, a modified Steiner stain was performed which demonstrated several spirochetes within the epidermis.

**Figure 2:**
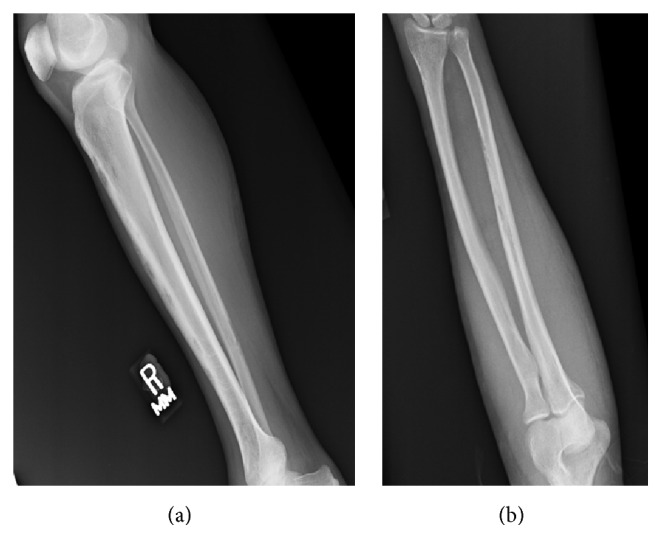
Plain radiographs of right tibia/fibula (a) and left forearm (b) showing multifocal region of cortical irregularities scattered throughout the diaphysis.

**Figure 3:**
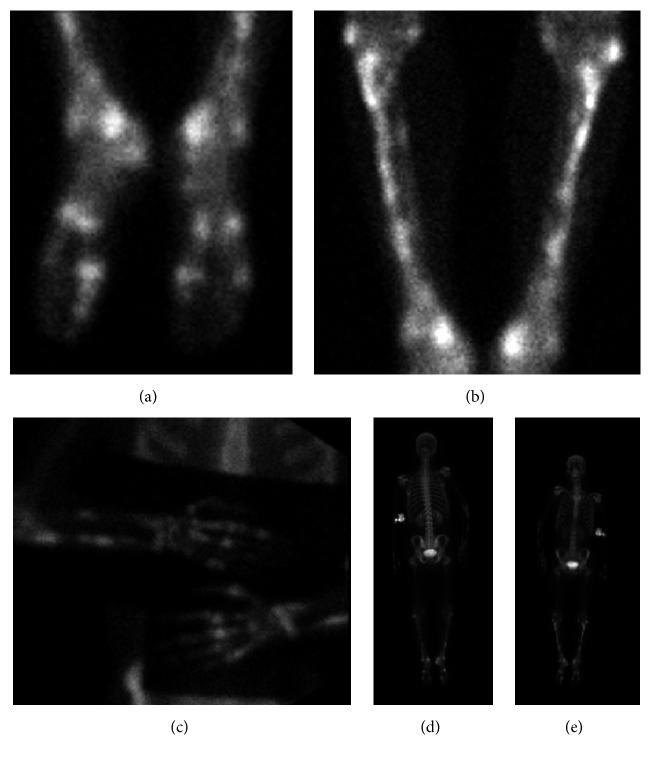
Nuclear medicine bone scan of the whole body showing extensive bilateral polyostotic patchy uptake within the distal appendicular skeleton, including the forearms, hands, tibias/fibulas, and feet. ((a) and (b)) Feet and tibiae, respectively. (c) Bilateral forearms and hands. ((d) and (e)) Whole body scans.

Plain radiography of the hands and feet revealed polyostotic cortical irregularities of the right foot and both hands, without joint involvement. Roentgenogram of the legs and forearms demonstrated cortical irregularities of the ulnar and radial diaphyses and of the tibial and fibular diaphyses, bilaterally (Figure 2). Roentgenogram of the skull was negative for bony lesions. Follow-up bone scintigraphy was impressive for bilateral, extensive polyostotic uptake within the hands, feet, forearms, and tibiae/fibulae (Figures 3(a)–3(e)).

Based on the positive serology and bone imaging findings, the patient was diagnosed with secondary syphilis osteitis and also now with acquired immunodeficiency syndrome (AIDS).

Given his penicillin allergy, the decision was made to treat the patient with an oral regimen: doxycycline 100 mg twice per day for a total of 6 weeks. He commenced receiving Atovaquone for* Pneumocystis jiroveci* prophylaxis. Prior to discharge (hospital course, day 8), a repeat RPR was performed, with a declining titer of 1 : 32.

**Figure 4:**
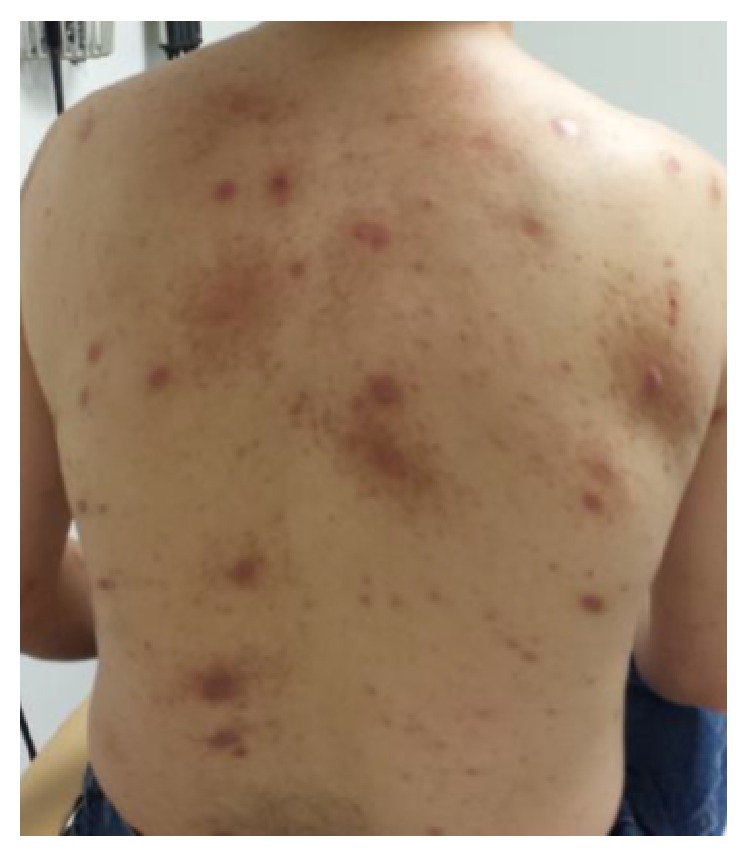
Photograph of the patient's back upon completion of treatment with doxycycline. Note dramatic improvement in lesions with few persistent areas of discoloration.

The patient was subsequently referred to a HIV clinic where he commenced antiretroviral therapy with Darunavir/Cobicistat, Dolutegravir, and Tenofovir/Emtricitabine. He had one additional follow-up with the HIV clinic and was noted to be compliant with the medications and tolerating them well. He was followed up by his primary care provider at 2 and 6 weeks. During and upon completion of the course of doxycycline, the patient noted considerable improvement in bone pains and joint swelling, which was corroborated on physical examination. At this time, the skin eruption was completely resolved (Figure 4). A follow-up blood count revealed an improvement in the cytopenias. The patient was referred for repeat imaging and RPR titers but defaulted on additional follow-up. Multiple attempts at communication were unsuccessful, and the patient is yet to return to either the HIV clinic or his primary care provider.

## 3. Discussion

Syphilis osteitis remains an uncommon manifestation of secondary syphilis, with the largest quoted case series being performed by Reynolds, who identified 15 patients with destructive bone lesions in 10,000 cases of early syphilis over a 21-year period (1919 to 1940) [6]. Another study performed by Thompson and Preston investigated skull involvement among 80 patients with secondary syphilis via conventional skull radiography. Seven patients had osteolytic lesions, with only 4 complaining of headaches [7]. Mindel et al. also performed a retrospective series spanning 20 years, with only 2 cases of syphilitic bone disease being diagnosed among 854 patients with secondary syphilis [8].

Secondary syphilis is associated with a disseminated stage and has varied clinical presentations, including mucocutaneous lesions, rash, and lymphadenopathy [9]. The skin rash in our patient was extensive and somewhat necrotic and has been described as “malignant” syphilis [5]. The presence of these clinical findings with concomitant bone pain or swelling prompted our investigation for syphilis osteitis. In syphilitic osteitis, examination may reveal tenderness over the involved bones, which is sharply localized and may be accompanied with local edema [10]. Additionally, pain has idiosyncratically been described to be exacerbated during the night and on exposure to heat [10, 11].

When spirochetemia occurs in syphilis, organisms can infect and involve the deeper vascular areas of the periosteum, with eventual extension into the Haversian canals and medullary spaces, resulting in periostitis, osteitis, or osteomyelitis [9]. Disease progression can develop into osteolytic or osteoblastic changes in the bones, often with predilection to the superficial bones [12, 13]. Orthodox radiography can occasionally demonstrate round areas with demineralization or sclerosis of the outer table and diploe, with less involvement of the inner table [7, 14]. Periosteal reactions are usually laminated or solid [15]. Generally, however, bone scintigraphy tends to be more sensitive than radiography in early detection of secondary syphilitic skeletal lesions and can be useful in ascertaining the extent of disease, guiding biopsy, and assessing response to therapy [16]. In a review performed by Park et al. of 37 patients with secondary syphilis and bone involvement, almost equal number of patients had disease affecting the limbs and skull, with multifocal lesions in 73% of cases. They also found that of the 13 bone lesions not detected by plain radiography 12 were detected by CT scan, MRI, and/or bone scintigraphy [4].

In addition to syphilis, multiple other etiologies should be considered in the HIV patient presenting with bone pain. Conditions more prone to the immunocompromised population include tuberculosis, pyogenic osteomyelitis, deep mycoses, and lymphoma, whereas non-HIV related conditions such as sarcoidosis or multiple myeloma are other possible differential diagnoses [17].

Identifying organisms on bone biopsy is inconsistent, with Halm reporting spirochete visualization by dark field microscopy in 50% of biopsied cases of bone involvement with early-stage syphilis [18]. Varied pathologic findings have been identified, including dense neutrophil infiltration without granuloma formation (Boone et al. [17]), perivascular infiltration with plasma cells and lymphocytes with some necrosis (Gurland et al. [19]), cortical and trabecular bone with attenuated inflammatory infiltrate composed largely of lymphocytes and plasma cells (Huang et al. [20]), bone necrosis with perivascular infiltration of plasma cells and lymphocytes and rare histiocytes (Kandelaki et al. [21]), and acute and chronic osteomyelitis with numerous treponemes seen on silver stain (Kastner et al. [22]).

Documentation of syphilis osteitis has remained confined mainly to case reports and review articles. To date, there have been no formal clinical trials to ascertain choice of antibiotic or duration of therapy to guide treatment of syphilitic bone disease. By convention, the usual treatment of secondary syphilis is with intramuscular benzathine penicillin G [23]. Though some authors cite satisfactory results with this approach [9, 24, 25], other authors favor more prolonged duration of therapy, such as penicillin G intravenously for either 4 weeks or 6 weeks [20, 26]. Another regimen undertaken by Fabricius et al. was Ceftriaxone 2 grams intravenously daily for 5 weeks in a patient with vertebral syphilis osteitis, achieving resolution of pain after the first week of treatment, and stable lesions on follow-up MRI [13].

In the review by Park et al., they noted that of 36 patients with secondary syphilitic disease more than 90% received treatment with penicillin (see Table 1). The median duration of therapy was 3 weeks. Antibiotic regimens most often prescribed were intramuscular benzathine penicillin G 2.4 mU weekly for 2 to 4 weeks, intravenous aqueous penicillin G 12–24 mU daily for 2 to 6 weeks, and intramuscular procaine penicillin G 0.6–1.2 mU daily for 10 days. In terms of outcome, 33 (92%) of patients had uneventful improvement solely with antibiotic therapy [4].

The conundrum arises for those patients who are intolerant of penicillin or are allergic. Alternative therapies include doxycycline and azithromycin. Boix et al. are the only authors thus far who have used a regimen devoid of penicillin to treat syphilis osteitis [27]. In that particular case, the patient had a history of Stevens-Johnson syndrome induced by oral amoxicillin. As such, a regimen consisting of oral doxycycline 100 mg twice daily for 16 weeks with the addition of oral azithromycin 1 gram daily for the first 10 weeks was employed. Follow-up bone scan showed resolution of all foci of increased uptake and repeat serology 6 months later showed more than fourfold decline in RPR titers. Because of the penicillin allergy and a history of poor follow-up, we elected to treat our patient with oral doxycycline for 6 weeks.

Rapid symptomatic relief is common following therapy; however osseous lesions can persist for up to seven to eleven months [18, 28]. Nonetheless, reports have cited that once appropriate therapy is completed, radiographic resolution of the boney lesions can be expected [6, 19]. Park et al. described a review of 10 patients who had follow-up bone scintigraphy performed between 1 and 11 months after treatment completion. Eight patients had complete or partial resolution of bone lesions, while 2 patients had unaltered imaging [4]. Our patient did not return for follow-up serology and imaging.

It is recommended that for HIV patients coinfected with syphilis nontreponemal titers (RPR) be repeated at 3, 6, 9, 12, and 24 months after completion of treatment [29]. A fourfold decrease by 6 to 12 months is considered an appropriate response [30]. Retreatment should be considered in the following scenarios: (a) objective clinical features of persistent or recurrent syphilis or (b) persistent or increasing nontreponemal titers [29]. Additionally, all probable or confirmed cases of early syphilis and all reactive nontreponemal laboratory test results should be reported to the local health department within one working day by public and private providers and laboratories [30].

## 4. Closing Remarks

In the preantibiotic era, the protean manifestations of syphilis were well described, including uncommon clinical features such as bone involvement in early disease [31]. However, following the advent of penicillin therapy, these exceptional presentations have become a rarity. With the ever growing HIV burden, there seems to be reemergence of these esoteric manifestations. In fact, over the past years, more than two-thirds of the cases of syphilitic bone disease were described among HIV patients [29].

Our patient presented with both syphilitic osteitis and severe cutaneous manifestations of “malignant” syphilis and was treated with doxycycline because of a penicillin allergy. This case highlights that, for those patients with a significant penicillin allergy or intolerance, alternate regimens consisting of doxycycline with/without azithromycin for an extended course can be considered.

## Figures and Tables

**Table 1:** Antibiotic treatment of 36 cases of secondary syphilis with bone involvement [4].

Antibiotic	Number (%) of patients
Penicillin	33
Benzathine penicillin G	19
Intravenous penicillin G	12
Procaine penicillin G	8
Other or unspecified penicillin regimens	4

Tetracycline	1

Doxycycline + azithromycin	1

Cephaloridine	1
